# Self-perception of voice, hearing, and general health in screening for voice changes in older women

**DOI:** 10.1590/2317-1782/20232022063en

**Published:** 2024-01-08

**Authors:** Maria Clara Rocha, Bárbara de Faria Morais Nogueira, Flávio Barbosa Nunes, Adriane Mesquita de Medeiros

**Affiliations:** 1 Graduação em Fonoaudiologia, Universidade Federal de Minas Gerais – UFMG - Belo Horizonte (MG), Brasil.; 2 Programa de Pós-graduação em Ciências Fonoaudiológicas, Universidade Federal de Minas Gerais – UFMG - Belo Horizonte (MG), Brasil.; 3 Departamento de Otorrinolaringologia, Universidade Federal de Minas Gerais – UFMG - Belo Horizonte (MG), Brasil.; 4 Departamento de Fonoaudiologia, Universidade Federal de Minas Gerais – UFMG - Belo Horizonte (MG), Brasil.

**Keywords:** Aging, Voice, Elderly, Presbyphonia, Voice Alteration, Self-perception

## Abstract

**Purpose:**

To verify the association between sociodemographic factors, vocal behavior, morbidities, and self-perception of voice, hearing, and general health in older women with voice disorders.

**Methods:**

The sample had 95 older women aged 60 to 84 years (mean of 69,5). They were interviewed with a structured questionnaire on sociodemographic aspects, health, and vocal behavior. The Screening Protocol for Voice Disorders in Older Adults (RAVI) was used to identify the presence of voice disorders.

**Results:**

Participants who had finished high school and were retired predominated. The number of older women with voice disorders according to RAVI was 46.3%. Physical sensations such as dry throat, throat clearing, and itchy throat were the most common complaints. The group of older women with voice disorders had worse self-perception of voice quality, hearing, and general health and a higher frequency of upper airway infections than those without voice disorders (p ≤ 0.05).

**Conclusion:**

The vocal self-assessment measured with RAVI was statistically associated with self-perception of voice quality, hearing, general health, sore throat, sinusitis, and respiratory allergies.

## INTRODUCTION

The advancement of science to prevent and treat diseases responsible for mortality has improved living conditions worldwide, which explains the increase in older people’s life expectancy^(1)^. The concept of health goes beyond the absence of morbidities, focusing on the subject's autonomy and self-management. Physical, social, psychological, and demographic changes may occur throughout aging, reflecting on the voice, and impacting human communication^(2)^.

Voice disorders can be defined as problems in conveying verbal or emotional messages, characterized by flaws in the person’s voice or a perception that it is different from how it should be^(3)^. Changes such as vocal fold bowing, vocal process prominence, and spindle glottal gap may occur in natural aging^(4)^, possibly impairing the quality of voice.

Voice disorders in older adults may be due to laryngeal aging or have a functional or organic cause^(5)^. Such disorders in older people are mostly related to their physical, social, and behavioral health status^(6)^ and can be identified with a protocol named Screening for Voice Disorders in Older Adults (RAVI, in Portuguese), based on their self-assessed auditory-perceptual symptoms and vocal tract discomfort^(7-10)^.

Older people’s self-perception of vocal disorders is related to worse quality of life and restrictions in daily activities^(11,12)^. Healthy aging includes greater communication efficiency that promotes social interaction and maintains autonomy and well-being^(13)^.

Thus, the population’s increased life expectancy and need for good communication while growing older^(1,2)^ justify paying attention to voice care and identifying possible factors associated with older people’s voice changes to ensure them a better quality of life.

Therefore, this study aimed to verify the association between sociodemographic factors, vocal behavior, morbidities, and the self-perception of the quality of voice, hearing, and overall health in older women with voice disorders.

## METHODS

This cross-sectional, analytical, observational research was approved by the Research Ethics Committee under evaluation report number 83004518.5.0000.5149. Before beginning the interviews, participants signed an informed consent form.

The study had a convenience sample of 95 older women, aged 60 to 84 years. The inclusion criteria were being a female over 60 years old, not having a confirmed diagnosis of dysphonia, not having undergone speech-language-hearing therapy in the previous 12 months, and being able to answer the questions regarding these criteria. It is important to highlight that studies approaching both sexes indicate a higher prevalence of vocal disorders in women^(14,15,16)^.

Participants were recruited by inviting the community through printed material, with the research objective, inclusion and exclusion criteria, and the researcher’s email and telephone number. The interview was scheduled according to the older women's availability. A total of 158 older women agreed to participate in the research, although 63 did not attend the scheduled interview.

Data were collected at the Speech-Language-Hearing Functional Health Observatory at the Federal University of Minas Gerais (UFMG). Participants were interviewed with a structured questionnaire developed by the researchers on sociodemographic aspects (gender, age, education level, and retirement); health issues (self-perceived hearing, hearing aid use, self-perceived health, medication use, medical diagnosis of gastroesophageal reflux, respiratory allergies, sinusitis, and sore throat [more than three times a year], hypertension and diabetes), and vocal behavior (self-perceived voice quality, voice changes in the previous 15 days, smoking, and hydration). Medication use was assessed according to their name and intake. However, due to memory bias and the possible variety of medications they took, some older women were unable to answer this question. They were also asked whether a doctor had confirmed a diagnosis of any of the cited comorbidities – though biased by the difficulty in identifying whether they were acute or chronic. Voice disorders were screened with RAVI^(7-9)^.

The response variable in this study was defined through RAVI, a quick, easily applied, low-cost, non-invasive instrument that screens voice disorders in older people^(7)^. Its 10 questions have three answer options (no, sometimes, and always), ordered from 0 to 2 (no = 0, sometimes = 1, and always = 2). The analysis addresses the sum of all questions, and the questionnaire’s total score can vary from 0 to 20 points. Final scores higher than 2 points indicate a voice disorder, and those equal to or lower than 2 points indicate the absence of a voice disorder^(9,10)^.

After the collection, the data were organized in a dataset in Microsoft Office Excel and then analyzed in the Statistical Package for the Social Sciences – SPSS, version 21.0. All categorical variables were described in percentages, and the numerical ones, in measures of central tendency. The association between the presence or not of voice disorders and the other variables was verified with Pearson’s chi-square and Fisher’s exact statistical tests. The significance level was set at 5%.

## RESULTS

The measures of central tendency regarding age and RAVI results are shown in Table 1.

**Table 1:** Measures of central tendency of the age range and voice disorder (RAVI) (n = 95)

**Variables**	**Minimum**	**Maximum**	**Mean**	**Median**	**Standard Deviation**
Age	60.00	84.00	69.52	69.00	6.0
RAVI	0.00	11.00	3.01	2.00	2.8

There was a predominance of retired participants, aged 60 to 70 years, who had finished high school. None of the variables was statistically significant with voice disorders (Table 2).

**Table 2:** Frequency distribution of sociodemographic and vocal behavior data and comparison between older women with and without voice disorders (n = 95)

**Variables**		**Voice disorders**		
	**Total**	**Absent**	**Present**	
	**N (%)**	**N (%)**	**N (%)**	**P-value**
Age*				
60 to 70 years	57 (60.0)	32 (62.7)	25 (56.8)	
71 to 84 years	38 (40.0)	19 (37.3)	19 (43.2)	0.556
Education level*				
Illiterate	3 (33.2)	0 (0)	3 (6.8)	
Learned to read and write	16 (16.8)	8 (15.7)	8 (18.2)	
Middle school	17 (17.9)	7 (13.7)	10 (22.7)	0.083
High school	31 (32.6)	16 (31.4)	15 (34.1)	
Higher education	28 (29.5)	20 (39.2)	8 (18.2)	
Retirement**				
Yes	86 (90.5)	48 (94.1)	38 (86.44)	0.294
No	9 (9.5)	3 (5.9)	6 (13.6)	
Voice changes in the previous 15 days*				
Yes	11 (11.6)	5 (9.8)	6 (13.6)	0.560
No	84 (88.4)	46 (90.2)	38 (86.4)	
Smoking*				
No	64 (67.4)	35 (68.6)	29 (65.9)	0.778
Smoker/ex-smoker	31 (32.6)	16 (31.4)	15 (34.1)	
Hydration*				
Two or more liters	23 (24.2)	16 (31.4)	7 (15.9)	0.079
Less than two liters/never	72 (75.8)	35 (68.6)	37 (84.1)	

* Pearson’s chi-square test

** Fisher’s exact test

The frequency of symptoms reported in RAVI encompassed dry throat (65.3%), clearing of the throat (46.3%), itchy throat (25.2%), tired voice (20%), voice discomfort (14.8%), voice disappearing over the day (9.5%), voice worsening over the day (11.6%), effort to speak (14.8%), a burning sensation in the throat (12.7%) and pain in the throat (10.5%). Of the 95 elderly women, 44 had voice disorders according to RAVI.

Voice disorders were statistically significantly associated with the self-perception of voice, hearing, self-perception of health, sore throat, respiratory allergies, and sinusitis (Table 3).

**Table 3:** Frequency distribution of data on the self-perception of health and morbidities and comparison between older women with and without voice disorders (n = 95)

		**Voice disorders**		
**Variables**	**Total**	**Absent**	**Present**	**P-value**
	**N (%)**	**N (%)**	**N (%)**	
Self-perception of voice quality**				
Very good/good	66 (69.5)	43 (84.3)	23 (52.3)	
Average	25 (26.3)	8 (15.7)	17 (38.6)	<0.001^a^
Very poor/poor	4 (4.2)	0 (0)	4 (9.1)	
Self-perception of hearing*				
Very good/good	54 (56.8)	38 (74.5)	16 (36.4)	
Average	28 (29.5)	9 (17.6)	19 (43.2)	<0.001^a^
Very poor/poor	13 (13.7)	4 (7.8)	9 (20.5)	
Hearing aid use**				
No	90 (94.7)	47 (92.2)	43 (97.7)	0.368
Yes	5 (5.3)	4 (7.8)	1 (2.3)	
Self-perception of general health**				
Very good/good	58 (61.0)	37 (72.5)	21 (47.7)	
Average	28 (29.5)	13 (25.5)	15 (34.1)	<0.008^a^
Very poor/poor	9 (9.5)	1 (2.0)	8 (18.2)	
Medication use**				
Yes	89 (93.7)	47 (92.2)	42 (95.5)	0.683
No	6 (6.3)	4 (7.8)	2 (4.5)	
Gastroesophageal reflux*				
Yes	25 (26.3)	10 (19.6)	15 (34.1)	0.109
No	70 (73.7)	41 (80.4)	29 (65.9)	
Respiratory allergies*				
Yes	26 (27.4)	9 (17.6)	17 (38.6)	<0.022^a^
No	69 (72.6)	42 (82.4)	27 (61.4)	
Sinusitis*				
Yes	25 (26.3)	9 (17.6)	16 (36.4)	<0.039^a^
No	70 (73.7)	42 (82.4)	28 (63.6)	
Throat inflammations (more than three times a year)**				
Yes	6 (6.3)	0 (0)	6 (13.6)	<0.008^a^
No	89 (93.7)	51 (100)	38 (86.4)	
Hypertension*				
Yes	54 (56.8)	28 (54.9)	26 (59.1)	0.681
No	41 (43.2)	23 (45.1)	18 (40.9)	
Diabetes*				
Yes	19 (20.0)	11 (21.6)	8 (18.2)	0.681
No	76 (80.0)	40 (78.4)	36 (81.8)	

* Pearson’s chi-square test

** Fisher’s exact test

a p-value <= 0.05

## DISCUSSION

The RAVI result showed that 46.3% of participants had vocal disorders, a value close to that found in another study (44.5%)^(11)^ and lower than that found in the older population living in Natal, Brazil, whose prevalence was 51.4% (95% CI: 46.8-55.9)^(2)^. In older people living in long-term care institutions, this prevalence was 39.3%^(10)^. All studies mentioned above used RAVI to define voice disorders, but in different samples regarding social aspects, general health conditions, and recruitment.

Other studies measured voice changes in the general population over the age of 60 years with different instruments, finding them to range from 4.8% to 29.1%^(3,12,17)^. Methodological differences between the studies may explain result variations. However, voice symptom screening tends to identify a high prevalence of voice changes in older adults, which indicates the need to refer them for clinical evaluation.

The symptoms most reported by participants in the present study according to (RAVI) were dry throat^(2)^, phlegm, and itchy throat – a result similar to that in the literature^(10,17)^. These symptoms are believed to be related to inappropriate vocal behaviors (such as smoking and poor hydration), the effects of medication use, and upper respiratory tract infections (URTIs). Only the last aspect was statistically different between the groups with and without voice disorders.

Older people with voice disorders are more likely to report data on URTI comorbidities^(3,14,18)^. Voice disorders were common among older women in the present study who reported sore throats (more than three times a year) (n = 6, 13.6%), respiratory allergies (n = 17, 38.6%), and sinusitis (n = 16, 36.4%) in contrast with the group without voice disorders. Upper respiratory symptoms are associated with an increase in voice disorders due to inflammatory conditions and edema, which can affect the structures responsible for phonation^(15)^. Older people should be instructed about URTI recurrence and its impact on the voice and how to proceed to improve vocal health.

There was no statistical significance between hydration and voice disorders. However, 75.8% of older women reported drinking less than 2 liters of water per day. A study showed that more than 65% of adults aged 51-70 years in the United States do not meet hydration criteria. On the other hand, body hydration cannot be measured solely by investigating amounts of water. The assessment must consider the person's physical structure, weight, and health status^(19)^. Nevertheless, the proportion of older women with low water intake was high in the present study, despite the difficulty in adequately assessing hydration through an epidemiological survey and the lack of statistical significance.

Smoking is another important behavior to highlight. The number of older women who used or had previously used cigarettes was 32.6%. Although smoking was not statistically significantly associated with voice disorders in this or other studies^(12,16)^, evidence indicates that the larynx is the most sensitive organ to histopathological changes after exposure to cigarette smoke. Furthermore, prolonged exposure to smoke can cause voice quality impairment, vocal tract irritation, vocal fold edema, cough, burning sensation, secretions, and respiratory infections^(20)^. Moreover, other studies show the said association in this age group^(2,9)^. Smoking directly interferes with healthy aging, as it is aggressive to the vocal tract and the main risk factor for laryngeal cancer^(20)^.

Hydration and smoking behaviors can be changed and addressed by disease prevention and health promotion initiatives. The results of this study indicate the need for strategies to raise awareness of smoking cessation or reduction and the benefits of hydrating for older adults. They should also be instructed about the consequences of such inadequate habits on voice quality and when to seek a specialist for a better assessment of the voice and definition of diagnosis and management.

Medication use was reported by 89 (93.7%) participants in this research and was not statistically significant with voice disorders. However, almost all older women use medications, some of which have side effects that can affect the salivary glands and mucus in the respiratory tract and are considered a factor associated with voice disorders^(21,22)^. Older people frequently take medication to treat one or more chronic non-communicable diseases^(13)^. Most medications are not evaluated for their effects on the voice, and the interaction of medications prescribed for treatment may be related to physical and psychological effects that are difficult to identify^(21)^. Speech-language-hearing pathologists should be attentive to the possible side effects of medications on the voice and general health of older patients in clinical practice.

This study identified a high rate of hypertension (56.8%), though lower than that observed in another study (62.4%)^(12)^. Nonetheless, it was not associated with older adults’ voice disorders in either one. Hypertension is a highly prevalent disease among older people, affecting around 50% of them^(23)^, corroborating the results of this study. The use of specific medications for hypertension did not lead to differences in voice self-assessment and quality of life in hypertensive adults aged 54 to 87 years when compared to non-hypertensive individuals^(24)^. On the other hand, hypertensive individuals aged 24 to 34 years are more likely to develop voice disorders^(15)^. The interaction between older adults’ many chronic non-communicable diseases and other predisposing factors for voice disorders likely hindered the identification of a statistical difference.

There is scientific evidence of the relationship between self-perception of voice quality, hearing, and general health and self-assessment of voice disorders. The group of older women with voice disorders according to RAVI had worse self-perceived voice quality, hearing, and general health.

Most older women in this study who considered their voice to have an average (n = 17, 38.6%) or poor/very poor quality (n = 4, 9.1%) belong to the group with voice disorders, which indicates a greater perception of voice symptoms. As expected, it can be stated that older women who report more symptoms perceive greater changes in their voice quality, reinforcing the importance of the older patient's vocal perception in the process of screening voice disorders in clinical voice assessment.

Self-perception of hearing was also worse among older women with voice disorders – 43.2% (n = 19) reported it as average and 20.5% (n = 9) as poor/very poor. Older adults commonly have age-related hearing loss, which may cause oral communication and social interaction difficulties. A study showed that older adults’ perception of voice changes and participation restrictions due to hearing difficulties influence their quality of life^(13)^. Older people with the perceived impact of hearing problems on their daily lives were more likely to have voice disorders, possibly due to the need for auditory feedback to control voice production, with a direct influence on the adjustments they used^(24)^. Hence, the comprehensive clinical assessment of older adults to ensure better communication must include their perception of voice and hearing.

Furthermore, it is necessary to know how older people perceive their general health and how they experience the aging process. Among older women with voice disorders, 34.1% (n = 15) reported their self-perception of general health as average and 18.2% (n = 8) as poor or very poor. The worst reports of self-perceived general health were more frequent in the group with voice disorders. A study in 3,759 people older than 65 years found that perceiving a worse general health status increases the odds of having voice problems by around two times^(25)^. Therefore, multidisciplinary treatment is greatly important to improve the vocal condition of the older population^(26)^.

Older people’s self-perception of health status must be considered in their aging process. This information helps identify factors that may impair their quality of life. Thus, this research importantly contributes to the older population concerning their communication and age-related health issues.

A limitation of this study was the impossibility of identifying whether the symptoms and morbidities were acute or chronic at the time of the research. Therefore, the presence of acute conditions may have overestimated the prevalence of voice disorders in the study group. The possibility of memory bias in the older women’s interview answers must also be considered.

Although this study approached only older women, the literature does not report significant differences in the presence or absence of voice disorders defined with RAVI between sexes in older people^(2,10,11)^. However, further studies should seek to verify aspects related to voice aging in different sexes.

Thus, the results indicate the need for programs to promote health and prevent impacts on the voice with strategies that encourage changes in habits. The reduction of inappropriate habits, control of associated morbidities, and promotion of healthy aging provide better voice quality, greater social inclusion, physical well-being, and better health status for older people.

## CONCLUSION

The study results show a high presence of voice disorders in older women. Complaints related to physical sensations such as dry throat, throat clearing, and itchy throat were the most common. The self-assessment of voice disorders measured with RAVI was statistically associated with self-perception of voice quality, hearing, general health, sore throat, sinusitis, and respiratory allergies.
