# Supplementary material for: Self-perception of voice, hearing, and general health in screening for voice changes in older women
Source: Codas. 2024 Jan 8;36(1):e20220063. doi: 10.1590/2317-1782/20232022063en (PMC10782509; doi:10.1590/2317-1782/20232022063en)
Supplement: Translation

# Translation: Autopercepção da voz, audição e saúde geral no rastreio de distúrbio vocal em idosas

**DOI:** 10.1590/2317-1782/20232022063pt

**Authors:** Maria Clara Rocha, Bárbara de Faria Morais Nogueira, Flávio Barbosa Nunes, Adriane Mesquita de Medeiros

**Affiliations:** 1 Graduação em Fonoaudiologia, Universidade Federal de Minas Gerais – UFMG - Belo Horizonte (MG), Brasil.; 2 Programa de Pós-graduação em Ciências Fonoaudiológicas, Universidade Federal de Minas Gerais – UFMG - Belo Horizonte (MG), Brasil.; 3 Departamento de Otorrinolaringologia, Universidade Federal de Minas Gerais – UFMG - Belo Horizonte (MG), Brasil.; 4 Departamento de Fonoaudiologia, Universidade Federal de Minas Gerais – UFMG - Belo Horizonte (MG), Brasil.

**Keywords:** Envelhecimento, Voz, Idoso, Presbifonia, Distúrbio Vocal, Autopercepção

## Abstract

**Objetivo:**

Verificar a associação de fatores sociodemográficos, do comportamento vocal, morbidades e autopercepção da voz, auditiva e de saúde geral de idosas com distúrbio vocal.

**Método:**

Participaram 95 idosas com idades entre 60 e 84 anos (média 69,5). Realizou-se uma entrevista com apoio de um questionário estruturado contendo questões sociodemográficas, de saúde e do comportamento vocal. Utilizou-se o Protocolo para Rastreamento de Alterações Vocais em Idosos (RAVI) para identificar a presença de distúrbio vocal.

**Resultados:**

Houve o predomínio de participantes com ensino médio completo e aposentadas. O número de idosas que apresentaram distúrbio vocal segundo o RAVI foi de 46,3%. Queixas relacionadas às sensações físicas como garganta seca, pigarro e coceira na garganta foram as mais presentes. O grupo de idosas com distúrbio vocal apresentou pior autopercepção da qualidade vocal, audição, saúde geral e maior frequência de infecções de vias aéreas superiores quando comparadas àquelas sem distúrbio vocal (p≤0,05).

**Conclusão:**

Houve associação estatística entre a autoavaliação vocal mensurada pelo RAVI e a autopercepção da qualidade da voz, da audição, da saúde geral, inflamações de garganta, sinusite e alergias respiratórias.

## INTRODUÇÃO

O avanço da ciência na prevenção e tratamento de doenças responsáveis pela mortalidade proporcionou melhoria das condições de vida no mundo inteiro, o que explica o aumento da expectativa de vida da população idosa^(1)^. O conceito de saúde vai além da ausência de morbidades, sendo o principal enfoque a autonomia e autogestão do sujeito. Ao longo do envelhecimento podem ocorrer mudanças físicas, sociais, psíquicas e demográficas. Tais alterações podem ser refletidas na voz e desencadear impactos na comunicação humana^(2)^.

O distúrbio vocal pode ser definido como um problema na transmissão da mensagem verbal ou emocional, caracterizada quando o indivíduo apresenta falhas na voz ou sente que ela está diferente de como deveria ser^(3)^. No decorrer do envelhecimento natural, alterações como arqueamento das pregas vocais, proeminência dos processos vocais e fenda glótica fusiforme^(4)^ podem ocorrer e causar ou não prejuízos à qualidade vocal.

O distúrbio de voz em idosos pode ser decorrente do envelhecimento laríngeo ou de natureza funcional e orgânica^(5)^. A maioria dos distúrbios de voz em idosos está relacionado ao estado de saúde física, social e comportamental^(6)^. Para realizar a identificação de possível distúrbio vocal foi desenvolvido um protocolo denominado Rastreamento de Alterações Vocais em Idosos (RAVI), baseado na autoavaliação do idoso em relação a sintomas perceptivos-auditivos e de desconforto do trato vocal^(7-10)^.

A autopercepção de distúrbio vocal pelo idoso está relacionada a pior qualidade de vida e restrições nas atividades do dia a dia^(11,12)^. O envelhecimento saudável envolve uma maior eficiência da comunicação que propicia a interação social, manutenção da autonomia e sensação de bem-estar^(13)^.

Dessa forma, o aumento da expectativa de vida da população e a necessidade de se manter uma boa comunicação ao longo do envelhecimento^(1,2)^ justificam a necessidade de se atentar aos cuidados com a voz e identificar possíveis fatores associados às alterações vocais da população idosa a fim de favorecer uma melhor qualidade de vida.

Sendo assim, o objetivo deste trabalho é verificar a associação entre fatores sociodemográficos, do comportamento vocal, morbidades e autopercepção da qualidade da voz, auditiva e de saúde geral de idosas com distúrbio vocal.

## MÉTODO

Trata-se de uma pesquisa observacional analítica transversal, aprovada pelo Comitê de Ética em Pesquisa – COEP, sob número de parecer 83004518.5.0000.5149. Antes do início da entrevista, o Termo de Consentimento Livre e Esclarecido – TCLE, foi assinado pelas participantes.

O estudo foi realizado com amostra de conveniência de 95 mulheres idosas, com idades entre 60 e 84 anos. Os critérios de inclusão foram: idade acima de 60 anos, ser do gênero feminino, não ter diagnóstico confirmado de disfonia e não ter realizado fonoterapia nos últimos 12 meses e que foram capazes de responder as perguntas referentes a tais critérios. Ressalta-se que pesquisas realizadas com ambos os sexos, apontam maior prevalência de distúrbio vocal em mulheres^(14,15,16)^.

O recrutamento das participantes foi realizado por meio de chamada à comunidade e do uso de um convite impresso, contendo o objetivo da pesquisa, os critérios de inclusão e exclusão, e-mail e telefone de contato da pesquisadora. A marcação da entrevista foi realizada conforme a disponibilidade da idosa. O total de 158 idosas concordou em participar da pesquisa, no entanto, 63 não compareceram ao dia agendado para entrevista.

A coleta de dados foi realizada no Observatório de Saúde Funcional em Fonoaudiologia – OSF da Universidade Federal de Minas Gerais (UFMG). As participantes foram submetidas a uma entrevista com apoio de um questionário estruturado elaborado pelas pesquisadoras contendo as seguintes questões: sociodemográficas (gênero, idade, nível de escolaridade e aposentadoria); saúde (autopercepção da audição, uso de aparelho auditivo, autopercepção da saúde, uso de medicamento, diagnóstico médico de refluxo gastroesofágico, alergias respiratórias, sinusite, inflamações de garganta – mais de três vezes ao ano, hipertensão e diabetes) e comportamento vocal (autopercepção da qualidade vocal, mudança na voz nos últimos 15 dias, tabagismo, hidratação). O uso de medicamentos foi avaliado de acordo com o uso e nome, no entanto, devido ao viés de memória, como também a possibilidade de uso variado de remédios, algumas idosas não souberam responder essa pergunta. Quanto ao diagnóstico, as idosas foram solicitadas a informar se possuíam alguma das comorbidades apresentadas confirmadas pelo médico. No entanto, outro viés foi a dificuldade em identificar se essas doenças eram agudas ou crônicas. Para o rastreio do distúrbio vocal foi utilizado o protocolo RAVI^(7-9)^.

A variável resposta do estudo foi definida por meio da utilização do protocolo RAVI. Este instrumento permite rastrear o distúrbio vocal no idoso, além de ser rápido, de fácil aplicação, ter baixo custo e não ser invasivo^(7)^. Contém 10 questões com três possibilidades de respostas (não, às vezes e sempre) ordenadas de 0 a 2 (não=0 às vezes=1 e sempre=2). A análise é realizada por meio do somatório de todas as questões e a pontuação total do questionário pode variar de 0 a 20 pontos. Para indicação de distúrbio vocal o escore final deverá ser superior a dois pontos e para ausência de distúrbio vocal este deverá ser igual ou menor que dois pontos^(9,10)^.

Após a coleta, os dados foram organizados em um banco de dados no Programa Microsoft Office Excel. Posteriormente, foram analisados no Statistical Package for the Social Sciences – SPSS versão 21.0. Todas as variáveis foram descritas por meio de porcentagens (categóricas) e medidas de tendência central (numéricas). Verificou-se associação entre a presença ou não de distúrbio vocal e as demais variáveis por meio dos testes estatísticos Qui Quadrado de Pearson e teste Exato de Fisher considerando o nível de significância de 5%.

## RESULTADOS

As medidas de tendência central da idade e do resultado do RAVI se encontram na Tabela 1.

**Tabela 1:** Medidas de tendência central da faixa etária e distúrbio vocal (RAVI) (n=95)

**Variáveis**	**Mínimo**	**Máximo**	**Média**	**Mediana**	**Desvio Padrão**
Idade	60,00	84,00	69,52	69,00	6,0
RAVI	0,00	11,00	3,01	2,00	2,8

Verificou-se o predomínio de participantes na faixa etária de 60 a 70 anos, com ensino médio completo e aposentadas. Nenhuma das variáveis apresentou significância estatística com o distúrbio vocal (Tabela 2).

**Tabela 2:** Distribuição de frequência dos dados sociodemográficos e de comportamento vocal e comparação entre as idosas com e sem distúrbio vocal (n=95)

**Variáveis**		**Distúrbio vocal**		
	**Total**	**Ausente**	**Presente**	
	**N (%)**	**n (%)**	**n (%)**	**Valor P**
Idade*				
60 a 70 anos	57 (60,0)	32 (62,7)	25 (56,8)	
71 a 84 anos	38 (40,0)	19 (37,3)	19 (43,2)	0,556
Escolaridade*				
Analfabeto	3 (33,2)	0 (0)	3 (6,8)	
Alfabetizado	16 (16,8)	8 (15,7)	8 (18,2)	
Ensino fundamental	17 (17,9)	7 (13,7)	10 (22,7)	0,083
Ensino médio	31 (32,6)	16 (31,4)	15 (34,1)	
Ensino superior	28 (29,5)	20 (39,2)	8 (18,2)	
Aposentadoria**				
Sim	86 (90,5)	48 (94,1)	38 (86,44)	0,294
Não	9 (9,5)	3 (5,9)	6 (13,6)	
Mudança na voz nos últimos 15 dias*				
Sim	11 (11,6)	5 (9,8)	6 (13,6)	0,560
Não	84 (88,4)	46 (90,2)	38 (86,4)	
Tabagismo*				
Não	64 (67,4)	35 (68,6)	29 (65,9)	0,778
Fumante/ex fumante	31 (32,6)	16 (31,4)	15 (34,1)	
Hidratação*				
Dois litros ou mais	23 (24,2)	16 (31,4)	7 (15,9)	0,079
Menos de dois litros/nunca	72 (75,8)	35 (68,6)	37 (84,1)	

* Teste Qui.Quadrado de Pearson

** Teste Exato de Fisher

A frequência dos sintomas relatados no RAVI foram: garganta seca (65,3%), pigarro na garganta (46,3%), coceira na garganta (25,2%), cansaço na voz (20%), incômodo na voz (14,8%), sumiço da voz ao longo do dia (9,5%), piora da voz ao longo do dia (11,6%), esforço para a voz sair (14,8%), queimação, ardência na garganta (12,7%) e dor na garganta (10,5%). Das 95 idosas, 44 apresentaram distúrbio vocal segundo o RAVI.

Houve significância estatística na associação do distúrbio vocal com os seguintes aspectos: autopercepção da voz, audição, autopercepção de saúde, inflamações de garganta, alergias respiratórias e sinusite (Tabela 3).

**Tabela 3:** Distribuição de frequência dos dados de autopercepção de saúde e morbidades com a comparação entre os grupos de idosas com e sem distúrbio vocal (n=95)

**Variáveis**		**Distúrbio vocal**		
	**Total**	**Ausente**	**Presente**	
	**N (%)**	**N (%)**	**N (%)**	**Valor P**
Autopercepção da qualidade vocal**				
Muito boa/boa	66 (69,5)	43 (84,3)	23 (52,3)	
Regular	25 (26,3)	8 (15,7)	17 (38,6)	<0,001^a^
Muito ruim/ruim	4 (4,2)	0 (0)	4 (9,1)	
Autopercepção da audição*				
Muito boa/boa	54 (56,8)	38 (74,5)	16 (36,4)	
Regular	28 (29,5)	9 (17,6)	19 (43,2)	<0,001^a^
Muito ruim/ruim	13 (13,7)	4 (7,8)	9 (20,5)	
Uso de aparelho auditivo**				
Não	90 (94,7)	47 (92,2)	43 (97,7)	0,368
Sim	5 (5,3)	4 (7,8)	1 (2,3)	
Autopercepção de saúde geral**				
Muito boa/boa	58 (61,0)	37 (72,5)	21 (47,7)	
Regular	28 (29,5)	13 (25,5)	15 (34,1)	<0,008^a^
Muito ruim/ruim	9 (9,5)	1 (2,0)	8 (18,2)	
Uso de medicamento**				
Sim	89 (93,7)	47 (92,2)	42 (95,5)	0,683
Não	6 (6,3)	4 (7,8)	2 (4,5)	
Refluxo gastroesofágico*				
Sim	25 (26,3)	10 (19,6)	15 (34,1)	0,109
Não	70 (73,7)	41 (80,4)	29 (65,9)	
Alergias respiratórias*				
Sim	26 (27,4)	9 (17,6)	17 (38,6)	<0,022^a^
Não	69 (72,6)	42 (82,4)	27 (61,4)	
Sinusite*				
Sim	25 (26,3)	9 (17,6)	16 (36,4)	<0,039^a^
Não	70 (73,7)	42 (82,4)	28 (63,6)	
Inflamações de garganta (mais de três vezes ao ano)**				
Sim	6 (6,3)	0 (0)	6 (13,6)	<0,008^a^
Não	89 (93,7)	51 (100)	38 (86,4)	
Hipertensão*				
Sim	54 (56,8)	28 (54,9)	26 (59,1)	0,681
Não	41 (43,2)	23 (45,1)	18 (40,9)	
Diabetes*				
Sim	19 (20,0)	11 (21,6)	8 (18,2)	0,681
Não	76 (80,0)	40 (78,4)	36 (81,8)	

* Teste Qui.Quadrado de Pearson

** Teste Exato de Fisher

a Valor de p <=0,05

## DISCUSSÃO

O resultado do RAVI mostrou que 46,3% das participantes apresentaram distúrbio vocal, valor próximo ao encontrado em outro estudo (44,5%)^(11)^ e inferior ao encontrado na população idosa residente na cidade do Natal/RN na qual houve prevalência de 51,4% (IC95%:46,8-55,9)^(2)^. Em idosos residentes em instituições de longa permanência, essa prevalência foi de 39,3%^(10)^. Todos os estudos citados acima utilizaram o RAVI para definir o distúrbio vocal, porém com amostras distintas quanto aos aspectos sociais e condição de saúde geral, além das diferenças no recrutamento dos participantes.

Já em outros estudos, que mensuram as alterações vocais na população geral com idade acima de 60 anos com instrumentos diversos variaram de 4,8% a 29,1%^(3,12,17)^. As diferenças metodológicas dos estudos podem explicar a variação dos resultados encontrados, porém verifica-se uma tendência de o rastreamento de sintomas vocais identificar uma elevada prevalência de alteração de voz em idosos, que indica a necessidade de encaminhamento para a avaliação clínica.

Os sintomas mais relatados pelas participantes do presente estudo segundo o (RAVI) foram garganta seca^(2)^, pigarro e coceira na garganta, resultado semelhante ao da literatura^(10,17)^. Acredita-se que tais sintomas podem estar relacionados a alguns comportamentos vocais inadequados como pouca hidratação e tabagismo, assim como aos efeitos do uso de medicamentos e infecções de vias aéreas superiores (IVAS). Apenas o último aspecto se diferenciou estatisticamente entre os grupos com e sem distúrbio vocal.

Quanto aos dados de comorbidades de IVAS, idosos que apresentam distúrbios de voz são mais propensos a relatá-las^(3,14,18)^. No presente estudo, o distúrbio vocal foi frequente entre as idosas que relataram inflamações de garganta (mais de três vezes ao ano) (n=6, 13,6%), alergias respiratórias (n=17, 38,6%) e sinusite (n=16, 36,4%) quando comparado ao grupo sem distúrbio vocal. Sintomas respiratórios superiores estão associados ao aumento dos distúrbios vocais devido a condições inflamatórias e edema que podem afetar as estruturas responsáveis pela fonação^(15)^. Idosos devem ser orientados quanto a recorrência de IVAS e os impactos dessas morbidades para a voz e como proceder para melhorar a saúde vocal.

Não houve significância estatística entre a hidratação e o distúrbio vocal, no entanto, 75,8% das idosas relataram que ingerem menos de 2 litros de água por dia. Um estudo mostrou que mais de 65% dos adultos com idades entre 51-70 anos nos Estados Unidos não atendem aos critérios de hidratação. Porém, a hidratação corporal não pode ser mensurada somente por meio da investigação sobre a quantidade de água. A avaliação deve ser realizada de acordo com a estrutura física do indivíduo, levando-se em consideração o peso da pessoa avaliada e suas condições de saúde^(19)^. Então, mesmo diante da dificuldade na avaliação adequada da hidratação por meio de inquérito epidemiológico e da ausência de significância estatística verificada, foi elevada a proporção de idosas com baixa ingestão de água no presente estudo.

Outro comportamento importante de se destacar foi o tabagismo. O número de idosas que fazem e já fizeram uso de cigarro foi de 32,6%. Apesar do tabagismo não ter apresentado significância estatística ao ser associado ao distúrbio vocal, nesse e em outros estudos^(12,16)^, existem evidências de que a laringe é o órgão mais sensível às alterações histopatológicas após a exposição à fumaça do cigarro. Além do mais, a exposição prolongada à fumaça pode alterar a qualidade vocal, causar irritação do trato vocal, edema nas pregas vocais, tosse, sensação de queimação, secreções e infecções respiratórias^(20)^. Entretanto, outras pesquisas mostraram essa associação no grupo etário estudado^(2,9)^. O tabagismo interfere diretamente no envelhecimento saudável, sendo agressivo ao trato vocal e o principal fator de risco ao câncer de laringe^(20)^.

Os comportamentos de hidratação e de tabagismo são modificáveis e passíveis de ações de prevenção de doenças e de promoção da saúde. Os resultados deste estudo indicam a necessidade de desenvolvimento de estratégias quanto a conscientização da cessação ou redução do tabagismo e dos benefícios do hábito de se hidratar para a população idosa. Os reflexos de tais hábitos inadequados na qualidade vocal, devem ser orientados indicando ao idoso quando se deve procurar um especialista para uma melhor avaliação da voz e definição de diagnóstico e conduta.

O uso de medicamentos foi relatado por 89 (93,7%) participantes desta pesquisa e não teve significância estatística com o distúrbio vocal, entretanto, quase todas as idosas fazem uso de medicamentos. Determinados medicamentos causam efeitos colaterais que podem afetar as glândulas salivares e o muco das vias respiratórias e é considerado um fator associado aos distúrbios de voz^(21,22)^. É frequente o uso de medicamentos por idosos para tratamento de uma ou mais doenças crônicas não transmissíveis^(13)^. A maioria dos medicamentos não é avaliada quanto aos efeitos na voz, e a interação de medicamentos prescritos para tratamento pode ter relação com efeitos físicos e psicológicos difíceis de serem identificados^(21)^. Na clínica fonoaudiológica, deve-se ter atenção aos possíveis efeitos colaterais dos medicamentos sobre a voz e a saúde geral de pacientes idosos.

Foi identificado neste estudo o elevado relato de hipertensão (56,8%), inferior ao observado em outro estudo (62,4%)^(12)^, porém ambos não encontraram associação com o distúrbio vocal em idosos. A hipertensão é uma doença altamente prevalente entre as pessoas idosas acometendo cerca de 50% das pessoas nessa faixa etária^(23)^, corroborando o resultado deste estudo. O uso de medicamentos específicos para a hipertensão não promoveu diferenças na autoavaliação vocal e na qualidade de vida de adultos de 54 a 87 anos hipertensos quando comparados com os não hipertensos^(24)^. Por outro lado, indivíduos com hipertensão de 24 a 34 anos possuem maior probabilidade de adquirir distúrbios vocais^(15)^. Pode-se supor que o elevado número de doenças crônicas não transmissíveis com a interação com outros fatores predisponentes do distúrbio vocal em idosos tenha dificultado encontrar diferença estatística.

Foi encontrada evidência científica da relação entre a autopercepção da qualidade da voz, audição e da saúde geral com a autoavaliação do distúrbio vocal. O grupo de idosas com distúrbio vocal segundo o RAVI apresentou pior qualidade vocal, audição e saúde geral autopercebidas.

A maior parte das idosas deste estudo que considerou a qualidade vocal regular (n=17, 38,6%) e ruim/ muito ruim (n=4, 9,1%) pertencem ao grupo com distúrbio vocal, que indica uma maior percepção de sintomas vocais. Podemos dizer que idosas que relatam mais sintomas percebem maior alteração na sua qualidade vocal, como era esperado, reforçando a importância da percepção vocal do paciente no processo de rastreamento de distúrbio vocal em idosos e na avaliação clínica da voz.

A autopercepção da audição também foi pior entre as idosas com distúrbio vocal, sendo o relato de 43,2% (n=19) como regular e 20,5% (n=9) como ruim/muito ruim. Vale ressaltar que a perda auditiva associada à idade é frequente nos idosos e pode resultar em dificuldades na comunicação oral e interação social. Estudo evidenciou que as modificações vocais percebidas e a restrição da participação resultante das dificuldades auditivas influenciaram na qualidade de vida dos idosos^(13)^. Idosos com percepção de impacto no cotidiano ocasionado por problemas auditivos apresentaram maior predisposição à presença de distúrbio vocal, possivelmente pela necessidade do feedback auditivo para controle da produção vocal, com influência direta sobre os ajustes a serem adotados^(24)^. Com isso, conclui-se que a avaliação clínica integral do idoso para propiciar uma melhor comunicação deve considerar a percepção vocal e auditiva.

Além disso, é necessário conhecer a forma como os idosos percebem a saúde geral e como vivem o processo de envelhecimento. Dentre as idosas com distúrbio vocal, 34,1% (n=15) relataram a autopercepção de saúde geral como regular e 18,2% (n=8) como ruim ou muito ruim. Os piores relatos de autopercepção de saúde geral foram mais frequentes no grupo com distúrbio vocal. Estudo com 3.759 idosos acima de 65 anos encontrou que perceber pior estado geral de saúde aumenta em torno de 2 vezes a chance de apresentar problemas de voz^(25)^. Ressalta-se a importância do tratamento multidisciplinar para melhorar a condição vocal da população idosa^(26)^.

É importante salientar que a autopercepção das condições de saúde da população idosa deve ser considerada no decorrer do envelhecimento. Por meio dessas informações é possível identificar fatores que podem prejudicar a qualidade de vida desses indivíduos. Deste modo, esta pesquisa proporciona importante contribuição sobre a população da terceira idade, seja na perspectiva da comunicação, quanto nas questões de saúde que acompanham esse grupo etário.

Uma limitação do estudo é a impossibilidade de identificar se os sintomas e morbidades são quadros agudos ou crônicos no momento da pesquisa. Desta forma, a presença de quadros agudos pode ter superestimado a prevalência de distúrbio vocal no grupo estudado. Também deve-se reconhecer a possibilidade de viés de memória nas respostas fornecidas pelas idosas por meio de entrevista.

Apesar de este estudo ter sido realizado somente com mulheres idosas, diferenças significativas entre a presença ou não de distúrbio vocal definida pelo RAVI segundo o sexo de idosos não foi verificada na literatura^(2,10,11)^. Porém, novos estudos devem procurar verificar os aspectos relacionados ao envelhecimento da voz em diferentes gêneros.

Sendo assim, os resultados encontrados indicam a necessidade de programas de promoção da saúde e prevenção dos impactos causados na voz com estratégias que estimulem mudanças de hábitos. A redução de hábitos inadequados, controle das morbidades associadas e o favorecimento do envelhecimento saudável, proporcionam melhor qualidade vocal, maior inserção social, bem-estar físico e melhores condições de saúde da população idosa.

## CONCLUSÃO

Mediante os resultados deste trabalho concluiu-se elevada presença de distúrbio vocal em idosas. As queixas relacionadas às sensações físicas como garganta seca, pigarro e coceira na garganta foram as mais presentes. Houve associação estatística entre a autoavaliação de distúrbio vocal mensurada pelo RAVI e a autopercepção da qualidade da voz, da audição, da saúde geral, inflamações de garganta, sinusite e alergias respiratórias.
